# A collaborative integrated Indigenous knowledge-based flood risk reduction model

**DOI:** 10.4102/jamba.v17i1.1913

**Published:** 2025-10-09

**Authors:** Uchenna Omoruyi, Ogochukwu Nzewi, Vongai Mpofu

**Affiliations:** 1 Department of Applied Management, Administration and Ethical Leadership, Faculty of Management and Commerce, University of Fort Hare, Bisho, South Africa; 2 Department of Quality Assurance, Faculty of Science Education, Bindura University of Science Education, Bindura, Zimbabwe

**Keywords:** indigenous early warning, Western early warning, two-eyed seeing, Integration, preparedness, flood risk reduction

## Abstract

Research on integrating Indigenous and Western knowledge systems for disaster risk reduction is increasing, particularly in South African communities where flooding is a significant issue. An integrated approach combining these knowledge systems within flood early warning systems could enhance community preparedness and risk reduction strategies. However, a lack of thorough studies on this topic in Alice Town creates a gap in guidance for local governments. The absence of models for collaboration between local governments and communities further exacerbates this gap. This paper utilised an integrative Indigenous qualitative research methodology to generate data through community conversations, interviews with local authorities and policy analysis. Data were analysed using qualitative content analysis. Findings revealed that although Indigenous and Western early warnings are different, they are similar and should be integrated. So, a Collaborative Integrated Hybrid Flood Risk Reduction Model (CIHFRRM) was developed to guide the integration process for better early flood warning and align with the National Development Plan’s goal of enhancing local government’s responsiveness and sustainability. The study concludes that an Indigenous knowledge-based (IKS)-based integrated approach could provide new proactive opportunities for dealing with floods, thus increasing communities’ capacity to predict, monitor and be prepared to reduce damage or address potential threats of floods.

**Contribution:**

The study further recommends that the proposed model can be applied to other municipalities’ disaster plans in South Africa.

## Introduction

Several studies have researched integrating Indigenous and Western knowledge (WK) systems for disaster risk reduction (DRR) in local government. However, there is a lack of models showing how local governments, in collaboration with local communities, can integrate Western early flood warning systems with Indigenous knowledge (IK) of communities towards an effective early warning system (EWS) for flood risk reduction (FRR) in South African communities. A preliminary literature search shows South Africa does not have a mechanism to integrate IK with Western EWSs. Thus, we present a Collaborative Integrated Hybrid Flood Risk Reduction Model (CIHFRRM) based on research conducted in the Alice Community of the Eastern Cape, South Africa.

Like other parts of South Africa, Alice was recently hit by floods. The flooding extended to the neighbouring villages (Bramedo 2020). Alice’s flooding was the first of its kind in recent history and left a trail of destruction that brought the town to a standstill (Majangaza 2020). A young child was hospitalised after being hit by lightning, and a woman was injured after a building collapsed (Loubser 2020). In contrast to their rural counterpart, semi-urban areas like Alice seem alien to IK’s traditional practices. However, IK is possessed by different groups irrespective of their location. For instance, although Alice is semi-urban, some older people might retain IK sustained through migration to this small town.

Mercer et al. (2010) posit that practitioners usually underrate IK because it is considered inferior to Western science. To fully harness the value of IK, practitioners should combine it with Western science in addressing hazards and disasters, allowing the two systems to work together and strengthen one another (Dube & Munsaka 2018). The Indian Ocean tsunami illustrates how major disasters have stimulated attention towards IK and its incorporation alongside Western science in DRR efforts (Mallapaty, 2012). Combining the two knowledge systems reinforced their advantages, enhancing the effectiveness of DRR measures (Dube & Munsaka 2018).

Both national and international legislation recommend adopting an integrated approach to FRR, hereafter FRR. For example, *Section 42* of the *South African Disaster Management Act* places urgency upon government institutions managing disasters to cooperate and collaborate with various stakeholders in activities that address disaster risk (RSA 2002). This *Act* regards DRR as multisectoral, where the three spheres, the private sector, traditional leaders, communities and civil society, must cooperate and collaborate (RSA 2002). Of vital importance is that this legislation affirms the significance of IK as a key component of DRR. Specifically, Section 53 (2) (f) requires that a municipal area’s DRR plan consider IK of DRR (RSA 2002). The Constitution supports this perspective based on the provision of Section 152, which mandates municipalities to involve the participation of local communities in local government affairs (RSA 1996). Internationally, the Sendai Framework for Disaster Risk Reduction (2015–2030) seeks to strengthen the resilience of member states by encouraging broad-based collaboration among all stakeholders in creating a conducive environment that enables DRR (UNISDR 2015). One of its targets, ‘Substantially increase the availability of and access to multi-hazard early warning system (MHEWS) and disaster risk information and assessments to people by 2030’ (UNISDR 2015:9), is critical to the overall objectives of this study, which aims to provide a model for integrating Western and Indigenous early warning (IEW) systems for more effective availability and dissemination of disaster early warnings to at-risk communities. Moreover, in South Africa, as proposed by the Sendai framework, its successful implementation becomes vital to reducing losses of lives, properties, businesses and infrastructure as suggested by the Sendai framework. Although South Africa has developed a MHEWS to forewarn against disasters so that those at risk of impending disasters can prepare to mitigate the impact of such disasters, much remains to be desired. There is still the unavailability of and access to MHEWSs and risk information for people in local communities where many disasters occur in South Africa (Khoza, van Niekerk & Nemakonde 2022; Van Niekerk, Coetzee & Nemakonde 2020).

Therefore, this article proposes a collaboration model or framework integrating IEW with the Western system. Integrating these systems enhances community capacity to forecast, monitor and prepare for floods, mitigating damage and addressing associated risks (Tyubee 2021). The current system represents a top-down approach to early warning. This article argues that a community-based approach [bottom-up] through IKS can provide municipalities with an integrated grassroots approach to FRR.

### Theoretical framework

This research adopted two theories to argue for integrating Indigenous and Western early warnings (WEW) for flood reduction: Two-eyed seeing by Marshall and Bartlett (2004) and the Stakeholders theory by Freeman (1984).

### Two-eyed seeing

Two-eyed seeing is defined as ‘learning to see from one eye with the strengths of IK and from the other eye with the strengths of WK and using both eyes together, for the benefit of all’ (Bartlett, Marshall & Marshall 2012:295).

**FIGURE 1:**
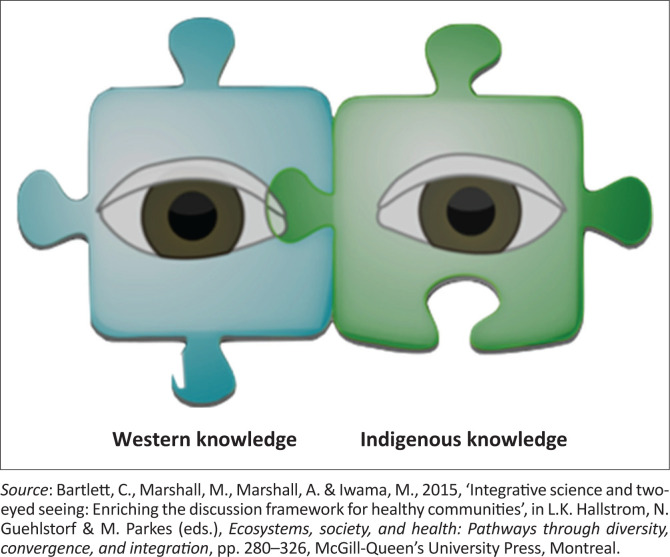
Two-eyed seeing.

Two-eyed seeing is represented by two eyes positioned behind two connected jigsaw puzzle pieces (Figure 1). The two connected puzzle pieces are knowledge systems, IK and WK, which must come together to create a holistic and complete solution to a problem (MacRitchie 2018). Each whole and distinct eye represents Indigenous and Western knowledge, respectively (Hatcher et al. 2009). Although different, both eyes are interconnected and should work together, like binocular vision (Bartlett et al. 2012). Therefore, in addressing flood risks through effective early warnings, two-eyed seeing acts as a guiding principle to highlight the strengths of IEWs through methods that focus on Indigenous ways of knowing and do the same for the Western method. This would create a balance between knowledge systems and worldviews by bringing together different knowledge and skills from each knowledge domain. So, just as the mobile nature of two eyes gives it the ability to look back and forth, we are thus able to weave back and forth between both knowledge systems (Bartlett et al. 2015) while drawing on their respective strengths as the situation may require.

Unarguably, these two knowledge systems are grounded in different paradigms that offer different world views; they must come together to form a comprehensive whole that can solve complex problems. Although this theory was chosen as a guiding principle informing the proposed integration of both EWSs, the researchers are cognisant of the Indigenous/Western dichotomy (Hatcher et al. 2009), and other authors cautioned researchers seeking to integrate both worldviews about the nature of knowledge from different perspectives. From the Western perspective, knowledge is a noun; it is singular and is passed objectively from one person to another. However, knowledge in the Indigenous worldview is a verb, and knowing is subjective. It is not a tool but a spirit, and the knowledge holders and users are not separate from it (Hatcher et al. 2009). With two-eyed seeing, the knowledge of the physical world is not distinct from the wisdom of the spiritual, and the accomplishments of Western science are blended with the wisdom of ancestors. Therefore, we have heeded this caution and understood that IK has elements of spirituality and that combining physical and spiritual elements makes IK whole (Battiste & Henderson 2005).

### Stakeholder’s theory

In 1984, Freeman defined stakeholders as any group or individual who can affect or is affected by attaining an organisation’s purposes (Freeman 1984:46). In 2004, Freeman and his colleagues updated this definition, describing stakeholders as those groups essential to an organisation’s existence and success (Freeman,Wicks & Parmar 2004). The stakeholder theory suggests that any private or public business operates within a complex network of multiple societal stakeholders (Steurer 2006). Moshodi, Coetzee and Fourie (2016) emphasised that businesses do not ‘exist in a vacuum’. An organisation’s financial and social success is influenced by the stakeholders impacted by its activities (Moshodi et al. 2016). According to this theory, effective management of stakeholder relationships contributes to higher financial returns and supports the enduring viability of firms (Moshodi et al. 2016). Therefore, the primary focus is to manage stakeholder relationships and interests to ensure the firm’s long-term success (Fontaine, Haarman & Schmid 2006).

Stakeholder management is not exclusive to the private sector but applies to the public sector. Local governments are institutions established in many urban areas of African nations. Their roles and responsibilities require them to work closely with various stakeholders, such as communities, civil society groups and non-governmental organisations, to fulfil developmental mandates. In South Africa, a local government is termed ‘developmental’ when it is committed to ‘work with citizens and groups within the community to find sustainable ways to meet their social, economic and material needs and improve the quality of their lives’ (RSA 1998).

According to Mojtahedi and Oo (2017), in DRR, stakeholders refer to individuals, organisations or entities that can influence, be influenced by, or perceive themselves as impacted by disasters. This includes any actor involved in managing disasters across the phases of prevention, response and recovery, as well as those whose interests may be negatively affected by such events. Communities, groups, organisations, institutions and the natural environment are typically seen as actual or potential stakeholders (Mojtahedi & Oo 2014).

This study acknowledges that individuals from diverse social statuses and cultural backgrounds live in communities vulnerable to flooding. We refer to these individuals as ‘flooding stakeholders’. Flooding stakeholders encompass all those directly or indirectly affected by natural hazards that cause flooding, and they stand to benefit from FRR efforts. Additionally, communities qualify as stakeholders because disasters directly impact them as the primary ecological overseers of their land. Thus, stakeholder theory emphasises the importance of involving the community in managing natural hazards. It suggests that risk reduction initiatives and interventions in Indigenous and urban communities would be more effective if they incorporated local knowledge systems.

Importantly, communities meet Freeman’s (1984) criterion of ‘can affect or is affected by’ and are directly impacted by disasters. Freeman (1984) argues that for organisations to be successful, they need the support of stakeholders. Therefore, the government must involve communities in DRR efforts to address vulnerabilities.

Effective stakeholder management is essential in DRR, as it enables entities to gain deeper insight into community risks while simultaneously promoting stronger trust, dialogue and engagement between stakeholders (Van Niekerk & Coetzee 2012). The end product is improved DRR. As a result, the potential to minimise disaster losses is considerably strengthened. This view is supported by Moshodi, Coetzee and Fourie (2016). They opine that because stakeholders determine the success or failure of any disaster risk intervention, stakeholder relationships are essential and should be managed to ensure a good relationship between stakeholders involved in achieving developmental mandates. Therefore, the government must involve communities collaboratively to address vulnerabilities when reducing flood risk. Thus, stakeholder theory illustrates the importance of consulting and involving communities and incorporating their knowledge systems into risk reduction initiatives and interventions.

### The conceptual framework

**FIGURE 2:**
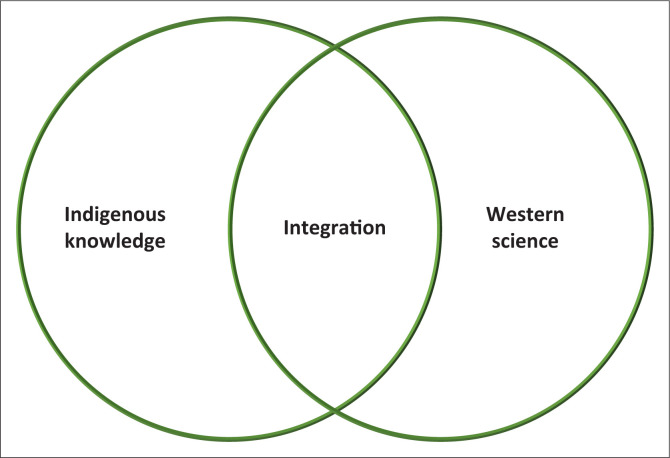
Conceptual framework.

Based on the foregoing theories, this section presents the main concepts that underpin this article. They are IK, Western science and integration. Figure 2 shows the interrelationship of these concepts in a conceptual framework. In the following section, we will present the definitions of these concepts and discuss how these concepts emerged from the two theories mentioned earlier.

On the one hand, ‘Indigenous knowledge is a network of knowledges, beliefs and traditions used to preserve, communicate, and contextualise Indigenous relationships with culture and landscape over time’ (Bruchac 2014:3). It is usually specific to a local area, shared by word of mouth, owned by the community together, holistic and changes over time to fit new situations (Mistry et al. 2020). It includes their knowledge, values, traditions, skills, culture and history and is still relevant today (Mji et al. 2017). According to Twigg, Indigenous knowledge is context-specific and social knowledge acquired from habiting in a particular locale over time which improves through new experiments, experiences, and external influences, and is orally communicated and intergenerationally transmitted (Twigg 2015). Based on the above, we define IK as the wisdom of a people, which is a vital tool for adaptation and survival based on the in-depth understanding of their natural environment, gained through intuitions, visions or transmitted through generations. Indigenous knowledge of disaster early warning is based on keen observation of the behaviours of birds, insects, vegetation, trees, animals, winds, air and water, temperatures, clouds, earth movements and celestial bodies (Habane 2010). This can be termed IEW. For this research, IEW is defined as traditional measures or practices used to forecast an imminent environmental hazard or disaster in communities, thus enabling preparedness against the effects of such threats.

On the other hand, Western science is the scientific knowledge and practices developed through Western scientific methods based on universal principles and empirical evidence. Western science is mainly developed in laboratories and can be documented by a qualified scientist. The term Western in the construct of Western science traces this form of science, philosophy and history to the global West. Thus, this Western descriptor implies that Western science is obtained from the Western world or hemisphere (Lwoga & Ngulube 2008) and differs from IK. Hence, scholars do not contend that IK differs from Western science. Still, they acknowledge their paradigmatic differences, such as Western science objectivity versus the subjectivity of IK and the physical sphere boundary of Western science versus the physical and spiritual connectedness of IK (Mpofu 2016; Odora Hoppers 2002). As the nation’s designated meteorological agency, the South African Weather Service (SAWS) is responsible for issuing Western early warning alerts, which are mandated to issue weather- and climate-related information and warnings about impending weather-related hazards such as floods. The SAWS utilises the South African Flash Flood Guidance System (SAFFG) to generate hydro-meteorological diagnostics, providing flash flood warnings with a lead time of up to 6 h derived from rainfall persistence estimates (Poolman 2015). South African Weather Service issues the warnings and disseminates them to the disaster management centres (National, Provincial and Municipal) and the relevant municipalities for readiness action [preparedness], such as the evacuation of vulnerable or at-risk communities in the face of such hazards (Poolman 2015).

Lastly, we define integration as combining two independent knowledge systems into one compatible entity that recognises each knowledge system’s common and unique aspects and is strengthened through the synergy of both knowledge systems. In the context of this study, stakeholders (the municipality and the community) work together by merging their knowledge systems on a joint problem-solving platform to find sustainable solutions to flood risks. This definition implies that integration is not an abstract concept but involves interaction between entities in the real world. These entities capture knowledge, habits, processes and practices. In this study, the product of such integration would be a hybrid EWS with elements of both knowledge domains. This hybrid knowledge system will produce an early warning whose combined effect will be greater than the sum of its individual effects. This definition espouses collaboration and partnership between the community and municipality, where neither party has unequal power relations.

### Emergence of the conceptual framework from the theories

The stakeholder’s theory underscores the importance of involving the community in environmental issues and managing natural hazards through their knowledge systems. The theory foresees potential in integrating stakeholder (Western and Indigenous) knowledge systems in risk reduction initiatives and interventions in Indigenous and urban communities. This emphasises communities and government collaborations in managing disasters to reduce their effect.

The conceptual model reflects this theory by acknowledging the importance of integrating the knowledge and perspectives of indigenous communities (IK) with those of Western scientific experts (WK) to create a more inclusive, effective and practical approach to DRR.

Two-eyed seeing emphasises the importance of seeing FRR initiatives from Indigenous and Western perspectives or lenses and bringing them together for a sustainable solution to flooding (Hatcher et al. 2009). These two knowledge systems are unarguably grounded in different paradigms that offer different world views. However, based on two-eyed seeing, they must be integrated to form a comprehensive whole that can solve complex problems. Also, integrating it with Western science is an innovative method that can cater for both urban and rural communities. This will involve moving back and forth between Indigenous and Western ways of being, knowing and doing (Bartlett et al. 2015) for a holistic and complete solution to environmental problems (MacRitchie 2018).

The conceptual model reflects this theory by positioning IK and WS as two distinct but complementary knowledge systems that can be integrated through a two-eyed seeing approach.

## Research methods and design

An integrative Indigenous qualitative research methodology, utilising primary and secondary sources, underpins the research approach. By blending Indigenous and Western research methods, data were generated from community conversations, interviews with local authorities and policy analysis. This study was conducted in the Alice community, Raymond Mhlaba Local Municipality of the Eastern Cape. The target population consisted of community members with IK of weather and municipal officials associated with DRR functions. Therefore, the snowball variant of purposeful sampling was employed to select information-rich participants (Patton 2002) from the community who were likely to have information on IK for FRR. Judgemental sampling was applied to select information-rich participants from the municipality to provide rich and credible data from those who could add value to the study. A total of 13 participants were chosen for the study. There were eight participants from the community, comprising four women and four men. They include (1) an IK expert and traditional healer and (7) community elders. We define elders in this study as 55 years and above. These are pseudo-named Elders 1–8. Also, (2) two senior management officials from the municipality and three disaster management and public participation officials. These are pseudo-named Municipal officials A–E. All participants were AmaXhosa (Xhosas) but held multicultural perspectives (religious, indigenous and western) on flooding and risk management through residing in a semi-urban locality and their religious faiths. Thus, generating data from the community and the municipality to determine their understanding of IK and their views on the possible integration of both knowledge systems contributed to the richness of this study’s data. Based on the two-eyed seeing theory, their unique understandings added value to this study. Findings were analysed using qualitative content analysis.

### Ethical considerations

Ethical clearance to conduct this study was obtained from the University of Fort Hare and the University of Fort Hare’s Research Ethics Committee (No. REC-270710-028-RA Level 01). This study obtained ethical permission from the University’s Research Ethics Committee (UREC) with reference number: NZE031SOMO01. The researchers duly ensured the ethical principles of informed consent, voluntary participation, confidentiality and anonymity throughout the data generation process.

## Results

This study aimed to integrate Indigenous and Western early warnings (IEW and WEW) for FRR. Therefore, understanding the similarities and differences between these two EWSs and the possibilities of integrating them was necessary because they are not the same and may not be handled similarly. Therefore, participants’ views were sought in order to achieve these aims.

### Differences between the warning systems

Participants revealed that these systems are grounded in distinct knowledge domains with different modus operandi. Also, it was found that those who forecast IEW, such as traditional healers or Izangoma, can also offer solutions on how the imminent danger can be averted, as seen in the following response:

‘One uses Western technology, while the other uses local signs. The Western forecast gives a general weather report but cannot provide a solution to an imminent danger. On the other hand, the Indigenous knowledge forecast predicts weather, foresees imminent danger, and explains how it can be prevented to reduce or avert damage.’ (Elder 1, Traditional healer, female)

Furthermore, the participants who argued that the EWSs are different based their arguments on how both were generated. They argued that an individual developed an IEW, while a computerised system developed its Western counterpart. They also argued that an IEW for Alice Town might not apply to the surrounding communities, unlike its Western counterpart, which would give forecasts that may apply to an entire province. See the following excerpts:

‘They are different because IEW forecasts weather by observing the sky, clouds, whirlwinds, birds and animal behaviours. They can tell you what the whirlwinds signify. They can tell whether it will be ordinary rain or not. With WEW, they forecast weather depending on technology and computerised appliances, which are sometimes incorrect.’ (Elder 4, Village elder, female)

And to another participant:

‘I say they are not similar because what might be forecasted for Alice by IEW may be flooding. However, according to the Indigenous knowledge forecast, the warning for Gaga Village, near us, could be Tornadoes. However, for WEW, the same forecast applies to all the surrounding areas.’ (Elder 7, Village elder, male)

Another difference was that:

‘IK has some distinct mystic or spiritual elements which make it different from its counterpart. Secondly, IEW is based on a belief system, while WEW is based on tested and proven scientific methods.’ (Elder 6, Village elder, female)

All the municipal participants, including senior management, also agreed with the assertions above that both EWSs from these knowledge domains differed. Their differences were also based on how they are generated because while WEW uses technology, IEW depends on natural signs like clouds, wind patterns and animal behaviours. See their following reflections:

‘IK is predicted locally by individuals with the gift of forecasting weather. In contrast, WS is predicted through systems, not individuals.’ (Municipal Official C, male)

To another Municipal Official:

‘They are different because IEWs are the handiwork of a person with the ability or gift to forecast weather, whereas a computerised system generates WEWs. You do not need a gift to generate WEW.’ (Municipal Official D, male)

### Similarities between both systems

Participants’ similar views of Indigenous and Western early warning (EW) systems draw these systems into an intersection function space. The two knowledge systems in that space fundamentally forecast weather as they alert communities of impending danger. They only differ in their ways of weather forecasting and knowledge transfer. These knowledge systems also share scientific elements, as illustrated in the following excerpt:

‘They are not different. IEW is informed by observations and signs developed and tested over time. It is just that IK does not document; however, it is a scientific process just like WEW because it is proven accurate and spot-on.’ (Elder 3, Village elder, female)

According to another participant:

‘Both systems are similar because they are both early warning systems but are generated differently. They serve the same purpose: to provide weather forecasts and warn of any imminent danger.’ (Conversations with Elder 8, Village elder, male)

Another participant elaborates further:

‘Although Indigenous and Western, they are similar because IK contains some elements of science, just like its counterpart. For instance, both knowledge systems can predict the weather based on high temperatures. For Western science, high temperatures cause water to evaporate from rivers, dams, and even the atmosphere to condense into clouds and precipitate back into the earth as rain. As indigenous people, we also believe that rain comes after very high temperatures to cool the atmosphere. Secondly, covering mirrors with blankets to reduce the impact of lightning has a scientific undertone. Mirrors contain atoms and electrons, so if they are not covered, they may attract lightning, causing damage.’ (Elder 6, Village elder, female)

### Possibilities of integrating both early warning systems

Two responses, for and against integration, emerged from the participants. The for-group, which this study has labelled the ‘Pro-integration group’, believed there was a possibility of integration, while the against group, which has been labelled the ‘Anti-integration group’, completely disagreed with such a possibility. To the ‘Pro-integration group’, integration was possible if only the municipality would first acknowledge and respect IK and its holders:

‘The two systems can be integrated if the municipality agrees to acknowledge and respect the knowledge systems of Izangoma [traditional healers] and work with them. Izangomas’ knowledge and abilities are for the community’s benefit. However, we [Izangoma] should be taken more seriously. For instance, in the case of the recent floods, I, as a Sangoma, saw it coming. I believe others also did. If there were a platform for knowledge or information sharing, we [Izangoma] would have spoken out and informed the municipality of the imminent danger. Moreover, because we forecast danger and proffer a solution, we would have worked with the municipality to ensure the threat was averted by doing whatever was required.’ (Elder 1, Village elder, female)

Other participants also believed that integration would be possible if the community and municipality worked together to address the stigma of backwardness attached to IK:

‘Integration is possible only if Western knowledge holders respect the other [Indigenous] knowledge system and see it as valuable enough to provide value to weather forecasts. However, we know that it will never happen because Indigenous knowledge is seen as backwards and outdated, especially in these days of civilisation and technology.’ (Elder 4, Village elder, female)

Still, regarding the possibility of integration, other participants like Elder 5 believed both systems could be integrated but could not explain how. According to him:

‘Yes, they can be integrated. I do not know how, but I can explain why they should be integrated. Western science has yet to discover all that the Indigenous people have found. For instance, cows jump in excitement when they sense the coming of rain, and the ants gather their food when they foresee floods. Western science has not been able to conceptualise these findings; hence, integrating both knowledge systems can provide an opportunity for the community and municipality to work together, which will have a meaningful impact on flood reduction.’ (Elder 5, Village elder, male)

The next group, whom the researchers have named the anti-integration participants, believed that both EWSs could not be integrated. This latter group were the municipal officials. One of the reasons was that they belonged to different knowledge domains and were generated differently. One of the Municipal officials’ responses is as follows:

‘No, they cannot be integrated. The reason is that they belong to different worldviews … they are too different. Indigenous knowledge is verbal and unwritten, while Western science is computerised technology. Both cannot be brought together.’ (Municipal Official C, male)

Others argued that integration would not be possible because a solution to disasters that includes IK is not feasible today. They saw IK as primitive and archaic and thought it should not form a part of modern-day discussions in an era of science and technology. Specifically, one of the officials said:

‘No, they cannot be integrated because we cannot talk of indigenous ways of forecasting weather today. Our parents used it because they did not have anything better; hence, they had to improvise to ensure survival. However, with the rate of modernisation and technology, going back to indigenous ways of weather forecasting is like going back to the Stone Age. We should be going forward, not backwards.’ (Municipal Official E, male)

More arguments on why integrating both EWSs would be impossible were that Alice was not a rural community but semi-urban and semi-rural. Also, they argued that Alice was composed of people who practised different religions, such as Christianity, Islam and the traditional religion. Consequently, such prospects would be feasible where most people are uneducated or illiterate and dominantly traditional worshippers. However, in a context such as Alice with the above background, although IK exists, it would not be respected, nor would the holders of such knowledge be taken seriously. According to an official:

‘You must understand that Alice is not 100% rural, so there are educated people here, and most people do not believe in the traditional religion. Here, there are Muslims, Christians, and others. Indigenous knowledge is rooted in diabolism and ancestral worship. My family and I are Christians, so we will not respect any initiative associated with Indigenous knowledge and Izangoma. I can say the same for other people.’ (Municipal Official B, Male)

## Discussion

Findings indicate that the significant difference between both forecasts is that, unlike the Western forecast, those who provide the IEWs in some cases can also offer solutions to the impending danger. A case in point is the issue of the Sangoma (Traditional Healer) who can act as an intermediary between the living and the spiritual world. According to her, she could consult her ancestors, who would give her directions on what to do, what libations to offer or what umsebenzi (traditional works/rituals) to perform to avert imminent danger. This finding resonates with literature that IK reveals the cultural value placed on ancestry and ancestors, which shapes their natural, cultural and ideological systems (Robison 2020).

The participants’ views on the scientific nature of IK are very insightful. The scientific nature of IK has been a bone of contention between proponents of both knowledge domains. However, most scholars have been able to change the narrative and prove that IK is scientific knowledge. According to Shahai (2013), IK results from generations of scientific work produced by communities and is just as valid and effective as Western, lab-based science. It is scientific because its methods of generation are empirical, systematic and experimental (Bohensky & Maru 2011).

Also, findings on the differences between both systems resonate with the literature, where, unlike Western EWSs, which utilise weather and climate models grounded in quantifiable meteorological data, Indigenous forecasting draws on biophysical indicators from the environment alongside spiritual approaches (Liang 2017; Ziervogel & Opere 2010). Additionally, a common thread runs through the participants’ responses. The common thread is how the IEWs are generated, which can be explained as the epistemology [origin or nature] of IEWs, differentiating them from Western science. In her paper, ‘Decolonising Climate Discourse and Legitimating Indigenous Wisdom: Toward an Ecosystemic Episteme’, Robinson advised that a vital factor in understanding indigenous worldviews and their relational ontologies is the logic that underlies how they are generated (Robinson 2020). Understanding this logic is critical when comparing IK and Western science because they apply opposing philosophies of self and the environment.

In terms of integration, an exciting aspect of the findings is that those who believed in integration were mostly from the community. In contrast, officials from the municipality opposed the integration agenda. The researchers found it interesting that the community were willing and open to integration and working with the municipality to find sustainable solutions to the challenge of flooding, but the latter saw no such possibility. According to the White Paper on Local Government (RSA 1998), a developmental local government works with the community to find sustainable ways to meet their needs and improve their quality of life (RSA 1998). How can the municipality work with the citizens or incorporate their knowledge systems when they are unwilling to meet them where they are? The basis of the stakeholder’s theory is a partnership alliance between stakeholders who have a common challenge and are willing to solve it jointly.

**FIGURE 3:**
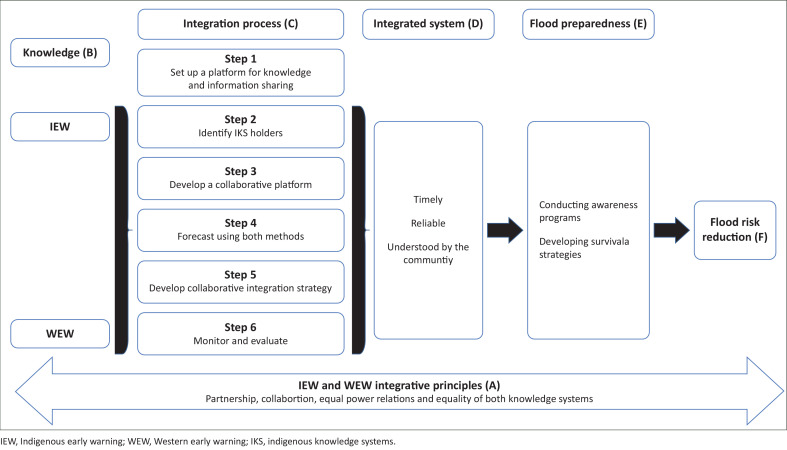
The collaborative integrated hybrid flood risk reduction model.

Thus, an essential part of the model is the need for a set of values or principles for such an integration strategy (Figure 3). This is because an integrated strategy cannot operate in an environment with profound scepticism about the value of IK to FRR. These principles include collaboration, equal power relations between parties and acknowledging the equality of both knowledge systems (see model principles).

Also, findings revealed that integration may not be possible because there is no relationship between IK practitioners and the municipality, and a platform has not been created in the community or by the municipality for informed and respectful interaction between IK holders and the municipality. The proposed model rationalises this concern into a practical IEW and WEW integration process that establishes a knowledge-sharing platform and identifies IKS holders.

In terms of findings on the differences between the two systems, these differences do not suggest that these two EWSs cannot be integrated. The similarities highlight that both EWSs are complementary and should be integrated to get the best out of both. Thus, intersections and similarities necessitate the development of a collaborative platform and a collaborative integration strategy, which have also been added to the IEW and WEW integration processes (see discussion on the collaborative integrated hybrid flood risk reduction model (CIHFRRM).

### The collaborative integrated hybrid flood risk reduction model

The study participants’ perspectives on the two EWSs and the possibility of combining them informed the development of this model, shown in Figure 3. The participants believed that integration would be possible if the municipality created a platform for mutual interaction and problem-solving with knowledge holders, acknowledged IK holistically and saw its value for DRR. So, although the community believed that both knowledge systems could be integrated, they did not know how; hence, this CIHFRRM seeks to guide future integration processes.

The CIHFRRM interacts with six main features, which are coded A–F for easy reference. It shows how IEW and WEW (B) can be integrated for effective flood reduction (F). As espoused by the two-eyed seeing theory, this integrative approach is essential for a holistic solution to FRR. This integration is to proceed through a six-step approach (C). It must be a collaborative venture of stakeholders. This aligns with the stakeholders’ theory, which posits that the community that is most affected during disasters must be involved in FRR initiatives to be effective. The integrative process yields an EWS (D) that is timely, reliable and well understood by the community. The level of preparedness (E) through awareness programmes and survival strategies determines the system’s effectiveness. All the activities are underpinned by IEW and WEW integrative principles (A) of partnership, collaboration, equal power relations and equality of knowledge systems. Each of these features is discussed briefly in the following section.

### Indigenous Early Warning and Western early warnings integrative principles

An essential part of the CIHFRRM is the need for a set of values or principles for such an integration strategy. This is because an integrated approach cannot operate in an environment with profound scepticism about the value of IK to FRR. These principles include partnership, collaboration, equal power relations between parties and acknowledging the equality of both knowledge systems. The basis of the stakeholder’s theory is a partnership and collaborative alliance between stakeholders who have a common challenge and are willing to solve it jointly. Power relations refer to the ability of persons or groups to control decision-making processes, knowledge creation and resources (Dekens 2007). Power relations can be within or between the community and outside organisations. Western knowledge practitioners may use terms such as ‘empowerment’ and ‘participation’ while undervaluing IK. Underestimating IK stems from perceiving it as archaic and its holders as illiterate (Liang 2017). Therefore, to eliminate power relations, all stakeholders involved in the integration strategy must recognise IK as a critical component of this integration that seeks to reduce flood risks in communities (Mercer et al. 2010). This recognition is necessary to see IK as equal to Western science to avoid a top-down implementation that discards and disrespects the opinions and communities’ opinions, and knowledge systems analysis of the frameworks also shows a limiting factor that cuts across the frameworks.

### Knowledge (B)

In line with two-eyed seeing, these are the two knowledge systems that must come together. Although both differ, especially in their forecasting method, they are also similar. They both forecast weather and alert communities of impending danger to reduce the flood risk.

### Integration process (C)

Step 1 is to set up a flood committee. This flood committee should comprise vital stakeholders such as community leaders and representatives, disaster management officials, ward committee members, community-based organisation representatives, volunteers, etc. The aim of the committee should be apparent. IK holders can provide critical weather information to the municipality in this space.

Step 2 is to identify IK holders. This process identifies IK experts of weather and flooding within the community to sit on the flood committee. These experts’ roles include providing and confirming the IK of early warning by the holders of the knowledge. The flood committee should engage these knowledge holders through workshops to create awareness of IEWs to the committee and also to bring the knowledge holders on board.

Step 3 is to validate the IK of the holders. This process involves confirming the IK of early warning by the holders of the knowledge. This could be practical tests, asking them to provide weather forecasts and verifying their accuracy. This process may take a while to give the IK holders enough time to provide accurate forecasts also bearing in mind the unpredictable nature of weather. Only those whose weather predictions are satisfactory may be selected.

Step 4 would be to forecast using both methods. This is the next stage in the process and should involve the provision of forecasts by the IK holders and the municipality. The purpose is to compare the similarities between both forecasts and their accuracy regarding results. If their results are similar and accurate, the next phase would be integrating efforts. This process should extend over a period to determine the accuracy and cater for changes in weather prediction.

Step 5 would be to develop a collaborative integration strategy. Integration in this research is the working together of the municipality and the community by blending their knowledge systems on a joint problem-solving platform to find sustainable solutions to flood risks. A collaborative, integrated strategy in this regard, therefore, is where both parties reach an agreement that weather forecasts from the selected IK holders through a specified channel would be acknowledged and acted upon. Such action could be comparing both forecasts and, if there is a cause for alarm, engaging the knowledge holders and members of the flood committee on what steps to take regarding the received forecast and the most suitable approach to disseminate the information to the community. The final decision must be a consensus between the community and municipality because the former is the actual recipient of imminent danger, the end-user of the early warning and would be more affected should it happen. Therefore, the communicated forecast will be a ‘Hybrid’ early warning, an output of different knowledge domains and a blend of Indigenous and WEW.

The sixth and final step would be periodically monitoring and evaluating the integration to determine whether its envisaged aims are achieved. All stakeholders involved in the integration must form a part of the monitoring and evaluation team so that their opinions on the successes or failures of the collaboration are documented. The product of the six steps discussed above was a collaborative integrated system the researchers coined, a hybrid EWS.

### The integrated system (D)

Three main features characterise the integrated hybrid EWS. These are timeliness, reliability and a system that the community understands. In support of the timely characteristics of the model, Poolman (2015) posits that an excellent early warning is only helpful if a warning is issued timeously to at-risk communities. A timely warning message must be given within a reasonable time to allow at-risk people to take preventive actions (Poolman 2015). The community’s understanding of the message is critical to the effectiveness of the warning message. This is because an understandable, reliable and credible message prompts actions (Jacks, Davidson & Wai 2010).

### Flood preparedness (E)

These elements of preparedness espoused by this model encourage proactivity by advising on conducting awareness programmes before a flood to prepare the community on survival strategies to strengthen their resilience should a flood occur.

### Flood Risk Reduction (F)

This is the envisaged output based on partnership, collaboration, equal power relations and equality of both knowledge systems.

## Conclusion

The CIHFRRM is a step-by-step process model developed from interactions with participants in this study on the possibilities of integrating both EWSs. This model, which seeks to guide future integration processes, shows how both EWSs for risk reduction can be integrated and how stakeholders seeking to integrate them can implement that process. Municipalities have a reactive approach to disasters because they allow disasters to happen before they react (Omoruyi 2024). Working with IK holders who can forecast an imminent weather-related disaster to initiate preparedness activities is critical. This is suggested as a joint project between municipalities, communities or other stakeholders who might find the model relevant. The study concludes that an IKS-based integrated approach could avail new proactive opportunities for dealing with floods, thus increasing communities’ capacity to predict, monitor and be prepared to reduce damage or address potential threats of floods. The study further recommends that the proposed CIHFRRM, once implemented, can be applied to other municipalities’ disaster plans in South Africa.
